# Economics of mental well-being: a prospective study estimating associated health care costs and sickness benefit transfers in Denmark

**DOI:** 10.1007/s10198-021-01305-0

**Published:** 2021-04-16

**Authors:** Ziggi Ivan Santini, Hannah Becher, Maja Bæksgaard Jørgensen, Michael Davidsen, Line Nielsen, Carsten Hinrichsen, Katrine Rich Madsen, Charlotte Meilstrup, Ai Koyanagi, Sarah Stewart-Brown, David McDaid, Vibeke Koushede

**Affiliations:** 1 The National Institute of Public Health, University of Southern Denmark, Studiestræde 6, 1455 Copenhagen, Denmark; 2 Parc Sanitari Sant Joan de Déu, Universitat de Barcelona, Fundació Sant Joan de Déu, CIBERSAM, Dr Antoni Pujadas, 42, 08830 Sant Boi de Llobregat, Barcelona, Spain; 3 ICREA, Pg. Lluis Companys 23, Barcelona, Spain; 4 Division of Health Sciences, Warwick Medical School, University of Warwick, Coventry, CV4 7AL UK; 5 Care Policy and Evaluation Centre, Department of Health Policy, London School of Economics and Political Science, London, UK; 6 Department of Psychology, University of Copenhagen, Øster Farimagsgade 2A, 1353 Copenhagen, Denmark

**Keywords:** Health economics, Healthcare utilization, Mental health, Well-being, Public health

## Abstract

**Background:**

Previous literature has examined the societal costs of mental illness, but few studies have estimated the costs associated with mental well-being. In this study, a prospective analysis was conducted on Danish data to determine 1) the association between mental well-being (measured in 2016) and government expenditure in 2017, specifially healthcare costs and sickness benefit transfers.

**Methods:**

Data stem from a Danish population-based survey of 3,508 adults (aged 16 + years) in 2016, which was linked to Danish registry data. A validated scale (WEMWBS) was used for the assessment of mental well-being. Costs are expressed in USD PPP. A two-part model was applied to predict costs in 2017, adjusting for sociodemographics, health status (including psychiatric morbidity and health behaviour), as well as costs in the previous year (2016).

**Results:**

Each point increase in mental well-being (measured in 2016) was associated with lower healthcare costs ($− 42.5, 95% CI = $− 78.7, $− 6.3) and lower costs in terms of sickness benefit transfers ($− 23.1, 95% CI = $− 41.9, $− 4.3) per person in 2017.

**Conclusions:**

Estimated reductions in costs related to mental well-being add to what is already known about potential savings related to the prevention of mental illness. It does so by illustrating the savings that could be made by moving from lower to higher levels of mental well-being both within and beyond the clinical range. Our estimates pertain to costs associated with those health-related outcomes that were included in the study, but excluding other social and economic outcomes and benefits. They cover immediate cost estimates (costs generated the year following mental well-being measurement) and not those that could follow improved mental well-being over the longer term. They may therefore be considered conservative from a societal perspective. Population approaches to mental health promotion are necessary, not only to potentiate disease prevention strategies, but also to reduce costs related to lower levels of mental well-being in the non-mental illness population. Our results suggest that useful reductions in both health care resource use and costs, as well as in costs due to sick leave from the workplace, could be achieved from investment in mental well-being promotion within a year.

## Introduction

The costs of mental health problems to society are substantial. In Europe, costs have been estimated to be more than 4% of its GDP—or over €600/$680 billion—across the 28 EU countries [1], with 1.6% accounted for by productivity losses due to mental health problems, 1.2% accounted for by higher spending on social security programs, and the remainder accounted for by direct expenditure on healthcare. Denmark incurs approximately USD $962.4 M in total direct costs, and $3.9bn annually in total indirect costs from mental health problems [2]. Although the issue of costs related to mental health problems has gained increasing recognition over the past 30 years [3], little is still known about the extent to which varying levels of mental well-being  across the entire (clinical as well as non-clinical) population influence costs (as opposed to focusing strictly on clinical populations, i.e. the costs of mental health problems or mental illness). According to a number of reviews [4–8], states of mental well-being have generally been found to be protective of physical as well and mental health and longevity, for example, through improved cardiovascular, immune and endocrine system functioning, reduced risk of heart disease, stroke and infection, better health behaviors, as well as enhanced resilience and recovery from illness, all of which may curb healthcare expenditure. Further, these reviews document that states of mental well-being have been shown to be beneficial in terms of productivity levels (e.g., better performance, reduced absenteeism) conducive to resilience and motivation to remain active on the labor market, as well as a range of social outcomes (e.g., enhanced sociability and prosocial behaviors) conducive to social capital and support, all of which may have implications for health and social service expenditure. However, while the costs of mental health problems have been documented in various evaluations, similar studies assessing the potential savings related to mental well-being are scarce.

**Fig. 1:**
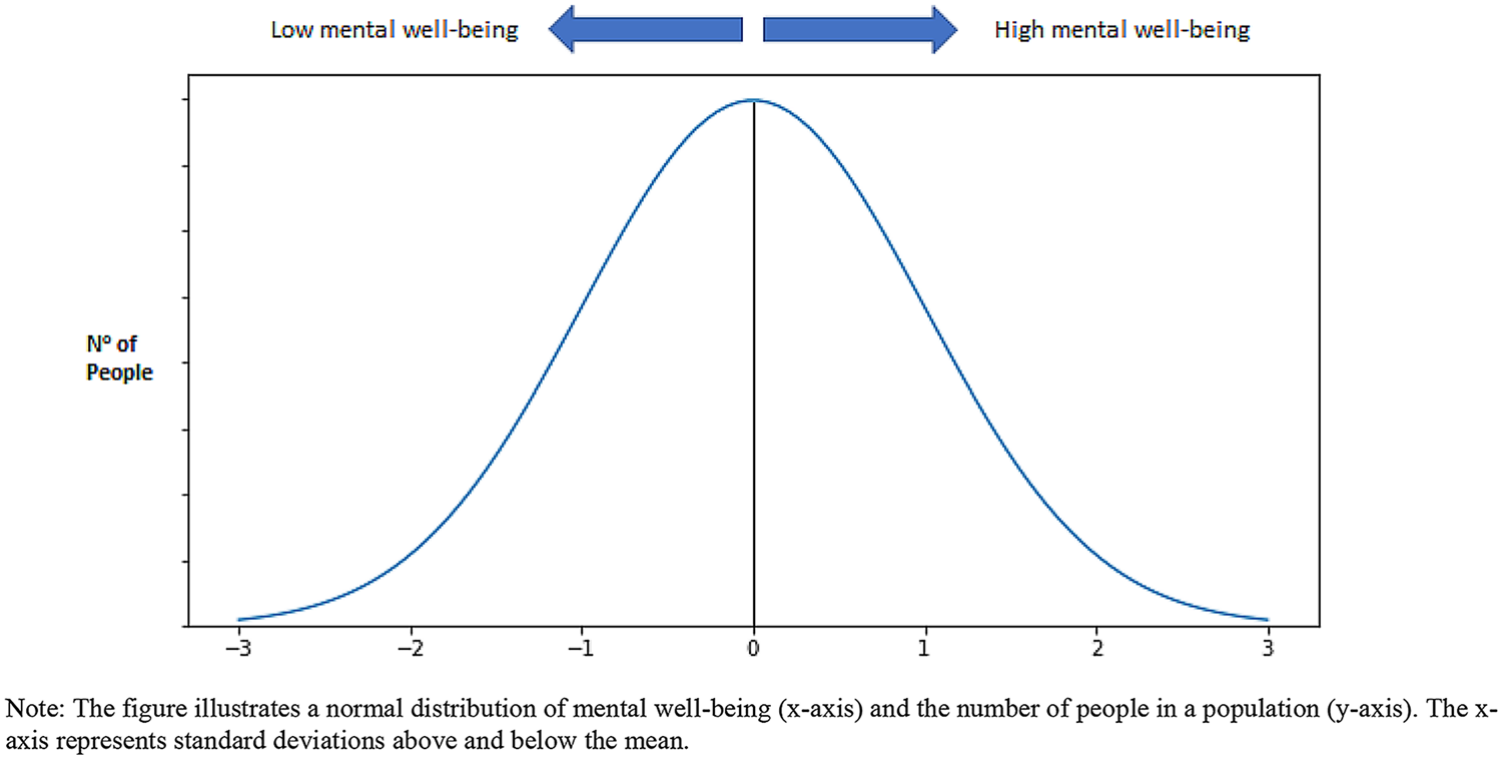
Conceptual figure of a mental health continuum based on a normal distribution of mental well-being

This study builds on a concept of mental well-being implicit in the Warwick–Edinburgh Mental Well-being Scale (WEMWBS), covering both hedonic (feeling good) and eudaimonic (functioning well) aspects, with both aspects being integral parts of the overall construct [9]. Functioning well includes growth and development and living in a way that brings meaning and purpose, a point which often seems neglected within health, medical, and epidemiological research. Eudaimonic aspects are developmental, tending to increase gradually over time; hedonic aspects are more influenced by fluctuations in the social and relational environment and may respond more rapidly to health promotion initiatives. High levels of eudaimonic well-being, in particular, are protective, offering the individual resilience to detrimental or stressful environments. Mental illnesses are diagnosed based on not feeling well and functioning poorly, positioning mental illness or lower levels of mental well-being at one end of a continuum, with higher levels of mental well-being at the opposite end (see Fig. 1). This also implies strong correlations between continuous measures of mental illness and mental well-being [10]. Importantly, conventional cost estimates for mental illness will not take into account the suffering of individuals with low mental well-being who may not meet diagnostic criteria [11]. Yet, lower levels of mental well-being may be associated with increased costs for individuals with and without diagnosed mental illness, and this may lead to considerable underestimation.

We identified seven studies that explored the predictive value of well-being measures specifically in regards to healthcare utilization/expenditure and loss to productivity [12–18]. All these studies were conducted on U.S. samples and based on economic outcomes generated from insurance data or survey self-report. Consistently, the results of these studies showed inverse associations between well-being and healthcare utilization and expenditure (e.g., hospital admissions, emergency room visits, prescription medicines) [12–18], productivity loss (unscheduled absence, disability leave, presenteeism, job performance) [12, 13, 17], and turnover (employee retention, voluntary and involuntary turnover) [12, 13]. These outcomes also displayed sensitivity to changes in well-being—that is, people whose well-being improved as a result of an intervention exhibited reduced healthcare use [13]. However, most of these studies were non-prospective investigations based on non-random samples [12, 15, 16]. Only two studies included nationally representative samples but were did not use prospective designs [17, 18], while two other studies were prospective but not based on random samples [13, 14]. No studies to date (to our knowledge) have reported a comprehensive analysis using register-based data from a European national setting in order to estimate associations between mental well-being and various cost outcomes. Such studies are strongly needed to advance knowledge about the potential financial savings that could be accrued by moving whole populations towards higher levels of mental well-being.

Studies which add to the evidence base of costs associated with different levels of mental well-being contribute to the ability of governments to secure financial sustainability. This is particularly pertinent as many countries and regions face budget constraints and may be inclined to cut funding for initiatives and interventions devoted to mental health promotion. Lack of empirical evidence on the economic benefits of mental well-being may explain why policy makers incline towards cutting funding for such initiatives. In this study, using a large sample of the adult Danish population, we assessed the association between mental well-being and government expenditure in Denmark, specifically healthcare costs and sickness benefit transfers. Based on the aforementioned evidence, we hypothesized that higher mental well-being scores (on a continuous scale) would be associated with lower healthcare costs and sickness benefit transfers in the following year.

## Methods

### Sampling

Our data came from The Danish Mental Health and Well-Being Survey 2016 (DMHWBS2016) [19], which is a random population-based sample of Danish men and women aged 16 years and above. The Danish government agency Statistics Denmark sent an electronic letter to the sampled individuals in October 2016 with information about the study and an invitation to participate. 3,508 people responded to the web-based survey (between October 18, 2016 and November 13, 2016) resulting in a response rate of 34%. Additionally, the survey was linked to the Danish Civil Registration System [20] via Statistics Denmark, which allows for the merging of data on employment status, household income, healthcare utilization, and social service use, among other variables. Each citizen in Denmark has a personal registration number, enabling linkage among registers [21]. All data are pseudonymized, so they cannot be traced back to specific participants. There is no formal agency for ethical approval of questionnaire-based survey studies in Denmark. The study complies with the Helsinki 2 Declaration on Ethics and is registered with the Danish Data Protection Authority; all confidentiality and privacy requirements were met. The participants’ voluntary completion and return of the survey questionnaires constituted implied consent.

### Outcomes: healthcare costs and sickness benefit transfers

All costs were extracted from Statistics Denmark for the year 2016 and 2017. This cost analysis utilized data from Danish national registers using each respondent’s anonymized civil registration number linked to the DMHWBS2016. Costs comprised (1) healthcare costs (general practitioners/specialists, hospitalizations, outpatient services, prescription medicines), and (2) costs in terms of sickness benefit transfers (including partial sickness benefit transfers). Unit costs for general practitioners and specialists are based on the current national health insurance rate [22]. Charges based on Diagnostic Related Groups (DRG) were used as unit costs for both costs of hospitalizations and outpatient services. Costs were omitted for healthcare services that did not involve treatment for illnesses, such as health services for contraceptive management (ICD-10 codes Z30) and other circumstances (ICD-10 codes Z76). For the costs of prescription medicines, public expenditure was calculated by subtracting user payments from the retail price of the medicine.

All healthcare costs were aggregated for: [1] the full year 2016, and [2] the full year 2017. Sickness benefit transfers (long-term absence, 31 + days) are estimated based on the weekly number of hours absent from work and respective salaries [23]. Sickness benefit transfers were also aggregated for: (1) the full year 2016, and (2) the full year 2017.

For a detailed description of cost components, see Appendix 1. All costs used for analysis are in 2016/2017 prices (DKK) and results were subsequently converted to international dollars (United States Dollars—USD, Purchase Power Parity—PPP) using an online conversion tool (2017 rates for price and target year, PPP values from the International Monetary Fund, 1DKK = USD$0.13 PPP) [24].

### Predictor: mental well-being

The Warwick–Edinburgh Mental Well-being Scale (WEMWBS) is a validated measure used to monitor mental well-being in the general population [25]. The scale has been validated in Denmark [10]. WEMWBS consists of 14 positively worded questions leading to a score between 14 and 70; the higher the score, the higher the mental well-being. WEMWBS scores were extracted from DMHWBS2016.

### Covariates

All sociodemographic variables were extracted from Statistics Denmark for the year 2016. The sociodemographic variables were: age; sex (female; male); migration background (Danish citizen; immigrant or descendent of an immigrant); marital status (married or registered partnership; divorced, terminated partnership or widowed; single); education (primary or unknown; youth education; tertiary education); employment status (employed; unemployed; student; retired; early retirement; other—employment status not defined); and income (lowest quartile; second-lowest quartile; second-highest quartile; highest quartile). Since 6.8% of data on income were missing, a ‘missing’ category was created for this variable.

Three variables pertaining to health status and health behaviors were included. The first two came from the register. To classify the presence of chronic conditions, we used the Charlson Comorbidity Index (CCI). It is based on 19 different medical conditions (myocardial infarction; congestive heart failure; peripheral vascular disease; cerebrovascular disease; dementia; chronic pulmonary disease; connective tissue disease; ulcer disease; mild liver disease; diabetes mellitus; hemiplegia; moderate/severe renal disease; diabetes mellitus with chronic complications; any tumor; leukemia; lymphoma; moderate/severe liver disease; metastatic solid tumor; AIDS), each weighted and assigned 1–6 points according to its potential impact on mortality, derived from relative risk estimates [26]. The CCI score is then categorized into three comorbidity levels: low (CCI = 0), medium (CCI = 1–2), high (CCI ≥ 3). To assess the number of psychiatric conditions, we added up the total number of psychiatric and self-injury diagnoses based on the ICD-10 codes F00-F99 (mental and behavioral disorders, including disorders relating to psychoactive substance use) and X60–X84 (intentional self-injury). The following variable came from the DMHWBS2016: level of physical activity was assessed with a binary variable (weekly or more; less than weekly). The sociodemographic and health status/behavior variables were included as covariates because they are associated with mental well-being and healthcare service utilization [13, 14, 18, 25, 27].

### Statistical analysis

STATA version 14 was used to perform all analyses. Following recommendations regarding the use of healthcare cost data characterized by heavily tailed and truncated distributions, we applied a two-part model [28, 29]. A two-part model is specifically designed to deal with limited dependent variables as we assume that a range of values may have a lower bound occurring in a large number of observations. In this case, individual annual healthcare expenditure and sickness benefit transfers may be zero if no event occurs. In the first part of the model, the probability of incurring any expenditure is calculated by a Probit model using the full sample. Then, a generalized linear model (GLM) with log link and a gamma distribution is fit for the subset of people that have any expenditure. More formally, the model can be written as the product of expectations from the first and second parts of the model, as follows:

$$E(y|{\mathbf{x}}) \, = {\text{ Pr}}(y \, > 0|{\mathbf{x}}) \times E(y|y \, > 0,{\mathbf{x}}).$$

Thus, the two-part model allows for a separate investigation of the effects of a predictor on the extensive margin (Probit model, if any expenditures) and on the intensive margin (GLM, amount of expenditures if any). Subsequently, the incremental effects of the predictor on the outcome for the combined probit and GLM are calculated [30].

The statistical analyses conducted were as follows. First, simple unadjusted graphs (fitted lines) were drawn to illustrate the relationship between the continuous WEMWBS score in 2016 and costs in 2017. Subsequently, the associations between the continuous WEMWBS score in 2016 and costs in 2017 were estimated adjusting for covariates. For each analysis estimating costs, we conducted a model 1 that adjusted for age, sex, migration background, marital status, education, employment status, income, and 2016 costs, and a model 2 that adjusted for all the aforementioned variables as well as chronic conditions, the number of psychiatric conditions, and physical activity. Both these two-part models were performed using the continuous WEMWBS scale as the predictor. In analyses where the outcome was sickness benefit transfers, we did not adjust for employment status in either model 1 or model 2, since sickness benefit transfers imply absence from employment or education. Also, since this outcome only pertains to individuals that were or would otherwise have been active on the labor market, the sample was restricted only to the working-age population, i.e., 16–64 years old (*N* = 1839).

All variables were entered into the models as categorical, except for age and the number of psychiatric conditions, which were continuous. In all analyses, a survey non-response statistical weight [19] based on age, education, region, marital status, employment status, and migration background was taken into account to attenuate selection bias. Both models were based on the sample with no missing data, and missing data were as follows: mental well-being 174 (5.0%); sex 0 (0%); age 10 (0.3%); migration background 0 (0.0%); marital status 10 (0.3%); education 0 (0%); income (see section on covariates); employment status 7 (0.2%); chronic conditions 0 (0%); number of psychiatric conditions 0 (0%); physical activity 5 (0.1%); healthcare costs in 2016 0 (0.0%); healthcare costs in 2017 0 (0.0%); sickness benefit transfers in 2016 0 (0%); sickness benefit transfers in 2017 0 (0.0%). In order to assess the influence of multicollinearity, we calculated the variance inflation factor (VIF) value for each independent variable. All VIFs were < 5, which is much lower than the commonly used-cut off of 10 [31], indicating that multicollinearity was unlikely to be a problem in our analyses.

## Results

All results that included costs were converted to USD PPP and are presented as such in the main tables, while the original results expressed in the Danish currency DKK are shown in Appendix 2. Information regarding the sociodemographic distributions of the study sample are shown in Table 1. The mean age of the study population was 52.1 years, with 54.2% of the participants being female.

**Table 1:** Characteristics of the 3,508 participants in the Danish Mental Health and Well-being Survey 2016

Characteristic	Category	*N*	%
Sex	Female	1852	54.2
Age	16–25 years	319	15.8
	26–44 years	735	28.8
	45–64 years	1437	32.1
	65 + years	1017	23.4
Migration background	Danish	3272	87.4
	Immigrant or descendent of immigrant	236	12.6
Education	Primary or unknown	831	33.9
	High school/Youth education	1457	39.2
	Tertiary education	1220	26.9
Marital status	Married/Registered partnership	1992	45.7
	Divorced, separated partners, widowed	589	17.3
	Unmarried	917	37.1
Income	Highest quartile	817	19.5
	Second-highest quartile	818	23.1
	Second-lowest quartile	818	26.5
	Lowest quartile	817	30.9
	Missing	238	6.8
Employment status	Employed	1906	51.0
	Unemployed	147	5.1
	Student	312	15.1
	Retired	948	21.8
	Social pension/Early retirement	120	3.6
	Other	68	3.4
Chronic comorbidity index (CCI)	Low (CCI = 0)	3309	95.2
	Moderate (CCI = 1–2)	173	4.1
	High (CCI ≥ 3)	26	0.7
Number of psychiatric conditions [mean (SD)]		0.3 (1.1)	
Physical activity	Weekly or more	3151	88.3
	Less than weekly	352	11.7
Mental well-being, range 14–70 [mean (SD)]		52.8 (8.4)	
Healthcare costs 2016 [median]^a^		519.6	
Costs of sickness benefit transfers 2016 [median]^a^		1968.6	
Healthcare costs 2017 [median]^a^		575.5	
Costs of sick benefit transfers 2017 [median]^a^		2947.2	

Data are unweighted n (weighted %) unless otherwise specified

^a^ All costs are in $PPP. Zero-costs were omitted

**Fig. 2:**
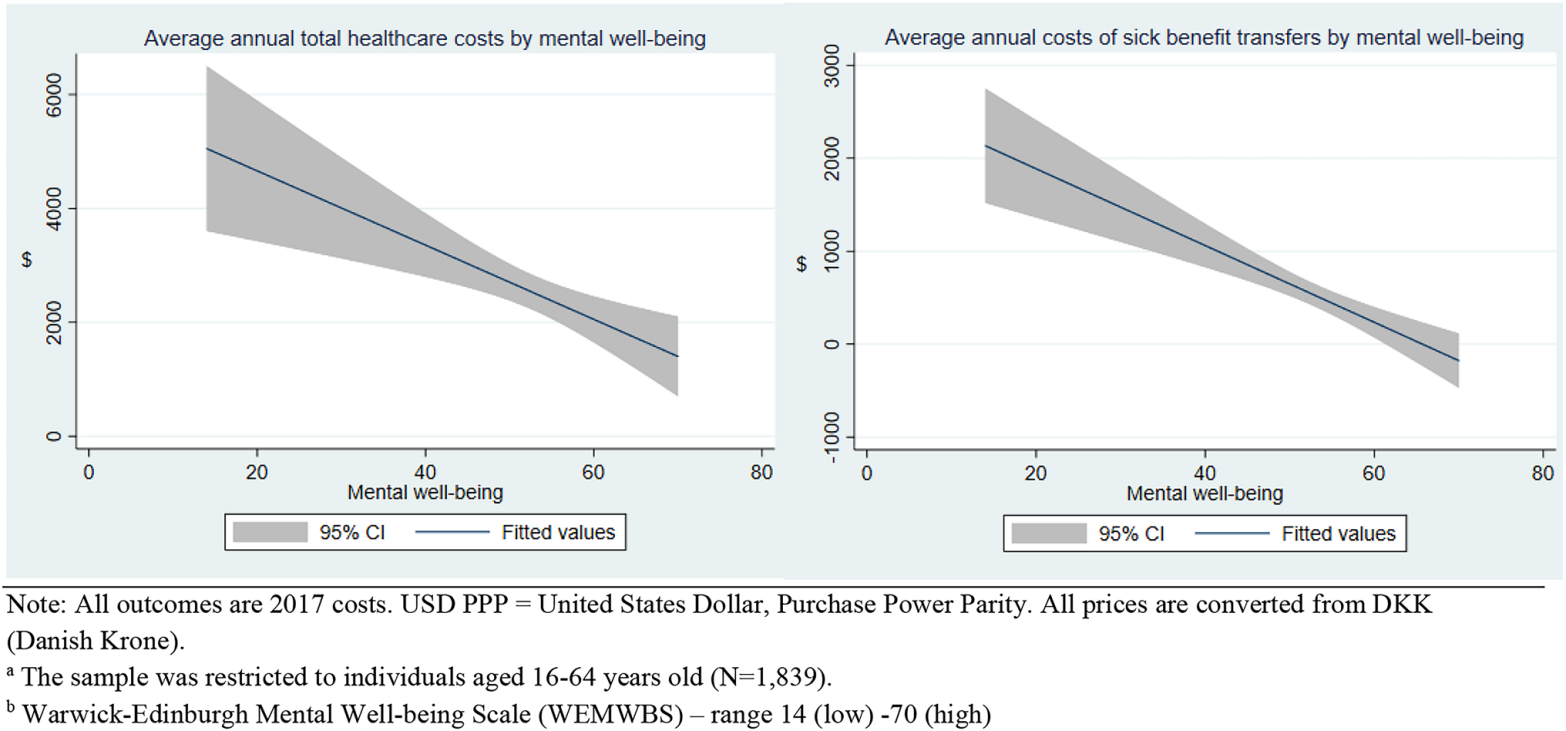
Unadjusted fitted lines for healthcare costs and sickness benefit transfers^a^ in 2017 (reported as USD PPP) by mental well-being^b^ (measured in 2016) among Danish adults 16 + . N

Figure 2 shows the unadjusted curves (fitted lines) of costs in 2017 by mental well-being measured in 2016 (continuous WEMWBS scale). The figures depict a downward trend in costs with higher levels of mental well-being.

For the analytical statistics, only model 2 results are reported here (both model 1 and model 2 results are shown in the tables). Table 2 shows the adjusted association between mental well-being (continuous WEMWBS scale) and per person healthcare costs and sickness benefit transfers. Each point increase in mental well-being measured in 2016 was associated with lower healthcare costs ($− 42.5, 95% CI = $− 78.7, $− 6.3) and lower costs in terms of sickness benefit transfers ($− 23.1, 95% CI = $− 41.9, $− 4.3) per person in 2017.

**Table 2:** Marginal effects of a one-point change in mental well-being (measured in 2016, continuous scale^a^) on healthcare costs and sickness benefit transfers (2017 costs, reported as USD PPP) per person among Danish adults aged 16 + years

	Model 1		Model 2	
	Marginal effect	95% CI	Marginal effect	95% CI
	Healthcare costs			
Mental well-being	− 41.9	− 74.8, − 9.1	− 42.5	− 78.7, − 6.3
	Sick benefit transfers^b^			
Mental well-being	− 28.8	− 48.2, − 9.3	− 23.1	− 41.9, − 4.3

USD PPP = International U. S. Dollars adjusted by Purchasing Power Parity. All prices are converted from DKK (Danish Krone)

Model 1 adjusted for age, sex, migration background, education, marital status, employment status (employment status not included for sickness benefit transfers), income, and 2016 costs

Model 2 adjusted for all the aforementioned covariates as well as chronic conditions, the number of psychiatric conditions, and physical activity

^a^Based on the 14-item Warwick–Edinburgh Mental Well-being Scale (WEMWBS), range 14–70

^b^The sample was restricted to individuals aged 16–64 years old (*N* = 1839)

## Discussion

In this study, we set out to estimate the extent to which continuous mental well-being scores in one year predicted healthcare costs/sickness benefit transfers in the subsequent year. Higher mental well-being (on a continuous scale) in 2016 was associated with lower healthcare costs and sickness benefit transfers in 2017. That is, after adjustment for a wide range of covariates including mental/chronic illnesses, physical activity, and costs in the previous year, each point increase in mental well-being was associated with lower healthcare costs ($− 42.5, 95% CI = $− 78.7, $− 6.3) and lower costs in terms of sickness benefit transfers ($− 23.1, 95% CI = $− 41.9, $− 4.3) per person in 2017.

### Contextualization of findings

In line with previous findings [12–18], our results confirmed our initial hypotheses by showing a pattern of decreasing costs with each point increase in mental well-being. This is a particularly strong finding, given that cost outcomes were generated in a way that took into account costs for the previous year, since these are known to be highly correlated with future costs [13, 14]. We adjusted for sociodemographics and a range of measures pertaining to health status and health behavior, which may influence differences in healthcare utilization and absence from work due to illness. Thus, our results suggest that the inverse relationship between mental well-being and costs is not accounted for by a specific vulnerable group of individuals that drive up costs due to being characterized by, for example, high levels of previous healthcare use, socioeconomic adversity or high levels of mental or somatic morbidity. Our results demonstrate that in addition to the well-known resource use and cost savings that can be achieved by preventing mental illness [32], considerable economic benefits due to better population health can be achieved by increasing mental well-being levels beyond the clinical or at-risk population. Our findings show that costs are continuously reduced with increasing levels of mental well-being, not just for levels above or below a particular threshold (as is generally the case in assessments of costs relating to mental illness), but for the entire distribution of mental well-being from lowest to highest. Thus, the greatest reductions in costs would be achieved when mental well-being is maximized among as many people as possible within a population.

Our results also suggest that improvements in mental well-being could generate a positive return on investment in the very short term. An intervention costing $65.6 (i.e., $42.5 + $23.1) per individual and generating a one-point increase in the WEMWBS scale would - by lowering the need for health care services and sick leave over the course of a year - reduce expenditure and free up scarce resources equivalent to the amount invested. Such assertions depend on evidence to show that mental well-being can be improved over a short time period, and the evidence base for this is now growing [13, 33–35]. Given that some stable healthcare costs attributable to chronic illness are amenable to improvement through mental health promotion, longer-term costs savings would be realized through mental health promotion interventions that deliver sustainable change.

### Implications for policy and practice

Denmark saw a dramatic 46% increase in public healthcare expenditure from 2000 to 2017 [36], with 16.4% of all public expenditure in 2017 spent on healthcare [37]. With escalating healthcare expenditures (which may occur for various reasons such as growing disease burdens as well as changes in budgets allocated to healthcare), there is an increasing need to identify factors that may drive down costs—in particular, modifiable factors that are predictive of health and disease, and by extension, healthcare utilization and costs. Previous research has already demonstrated that enhancing population levels of mental well-being is economically worthwhile in both the short and long term [38, 39]. Our results add to this evidence base and suggest that the promotion of population mental well-being—apart from being desirable in its own right—should have the additional benefit of curbing care costs for physical as well as mental illnesses, and that this could be achieved in a short time. Governments succeeding to do so would then have the freedom to potentially allocate these financial resources to other priorities within or beyond the healthcare sector. Similarly, funds that would otherwise have been transferred to workers due to sickness absence could then also be used in different welfare budgets or other purposes. Altogether, there is a need for policy makers to give much more priority to considering the potential social and economic impacts of population mental well-being. Rather than focusing discussions solely around healthcare delivery and insurance, policy makers would need to take measures to increase the number of individuals with higher levels of mental well-being, and through this process drive down healthcare utilization and lost productivity due to illness.

**Fig. 3:**
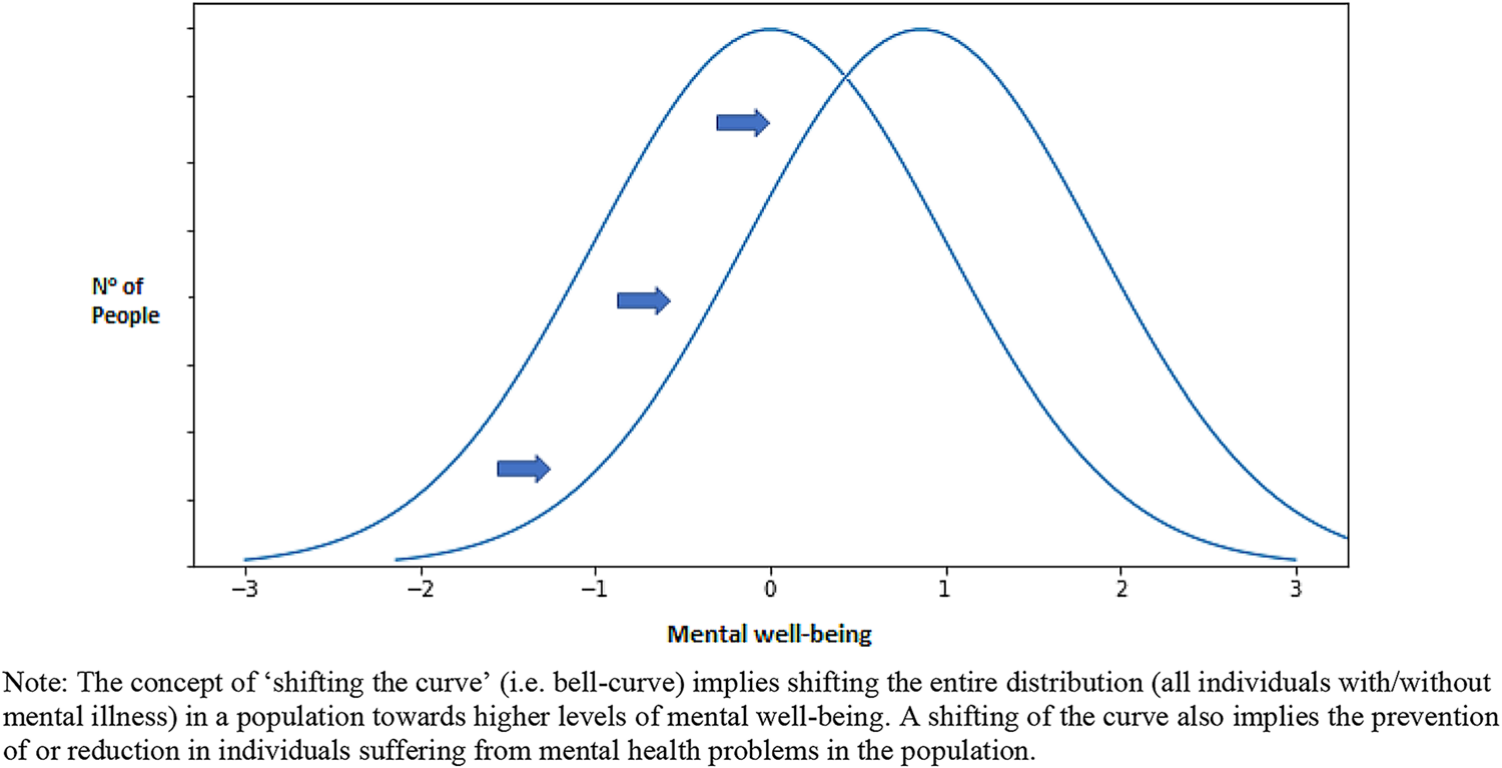
’Shifting the curve’ as an approach to population mental health

Mental well-being is modifiable, and supporting efforts to foster mental well-being throughout populations should be an end-goal in policy and practice [9]. Policy and research priorities formed by the European Commission and the World Health Organization support the view that a focus on promoting mental well-being is crucial for long-term growth and sustainable development [40–42]. In particular, a ‘shifting of the curve’ approach to population mental health is needed (see Fig. 3), which implies shifting entire population distributions towards higher levels of mental well-being [43].

Elsewhere, countries such as New Zealand have recently placed an emphasis on the promotion of well-being, particularly for younger people, in the country’s first-ever well-being budget [44]. Scotland monitors progress on indicators of activities that are likely to promote mental well-being such as proximity to green and blue spaces [45]. Recent research has shown that socioeconomic predictors of high mental well-being do not mirror those of low mental well-being [25, 46]. Hence, a mere focus on improving levels of education and wealth does not appear to be sufficient to promote higher levels of mental well-being. However, several relational and recreational factors predict both high and low mental well-being [47], suggesting that an additional focus on such factors would be especially relevant in universal mental health promotion initiatives.

Universal mental health promotion implies fostering behaviors and environments known to protect and enhance mental well-being. Such initiatives may encourage individuals to engage in mentally healthy behaviors, such as those that enhance awareness and self-regulation (e.g., concentration, flow, mindfulness, self-care), keeping active in various ways, maintaining contact with social ties and participating socially, as well as getting involved in meaningful challenges and causes. Initiatives may also support community organizations and municipalities in fostering environments that provide opportunities to engage in mentally healthy behaviors [48]. Importantly, promoting mental well-being universally not only helps to sustain individuals, but contributes to a ‘herd immunity’, where the more people that are characterized by higher levels of mental well-being in a community, the more likely it is that those with acute or long-term mental health problems can be supported [49, 50].

The latter point also applies in terms of healthcare utilization, as research has already demonstrated that higher community well-being (counties or zip codes with higher average well-being) is associated with reduced healthcare expenditure within those communities [16, 18]. Meta-analytic reviews further support the view that favorable psycho-social community environments are associated with better physical and mental health outcomes, as well as inversely associated with healthcare utilization [51]. One challenge in terms of developing an effective mental health promotion strategy is that policy makers may be inclined to look to the healthcare sector or to interventions devoted strictly to clinical populations as a means to address mental health. Yet, many initiatives that are likely to contribute to enhanced population mental well-being are often delivered outside of the health sector. Effective efforts would require inter-sectoral collaborations and a health-in-all-policies approach. Some promising areas for further research concern the value of investing in awareness-raising regarding mentally healthy behaviors [48, 52], protective environmental factors (e.g., green spaces) or culture/art as a way of promoting population mental well-being [32, 53].

Some strengths and limitations should be kept in mind when interpreting the results. Major strengths include the prospective design, the use of a validated scale for measuring mental well-being, and the use of a population-based survey linked with national registers. This approach made it possible to make direct links between mental well-being in one year and cost outcomes expressed in monetary terms in the subsequent year, as well as a range of register-based covariates. Also, since the outcomes and most covariates were from the register rather than from the survey, common methods as a result of single-source self-reported data are not an issue in this study. Some limitations are as follows: The response rate was 34%, and while this is not unusual for web-based surveys, selection bias cannot be ruled out. Response rates were higher for women, individuals aged 45 years old and above, individuals with higher education (tertiary), individuals who were married or in a registered partnership, employed individuals, individuals with a Danish (non-immigrant) background, and individuals with a higher income [19]. We have applied weights in all analyses, which has reduced statistical uncertainty to some extent. Some relevant variables were not available in our dataset, therefore, we could not adjust for factors such as alcohol consumption, smoking, and nutrition, although such factors may also be mediators. Finally, it may be observed that there was some overlap between the time of the survey (October 2016) and the data on costs (2016 and 2017).

In this study, our final results are based on analytical models where we adjusted for health status and health behavior (apart from demographics and socioeconomic factors). We did this to minimize confounding of health factors, but there is a possibility of overadjustment, since our models already took into account past healthcare utilization. It is also important to consider that healthcare costs and transfers are highly correlated from one year to the next due to ongoing illness. Some of these costs may respond positively to improvements in mental well-being, for example through improvements in self-care, health-related lifestyles, and greater resilience to stress. Our approach is conservative as it excludes all longer-term ongoing positive impacts on chronic illness, and instead focuses solely on the costs which are most immediately associated with mental well-being.

Some additional reflections should also be made in terms of the scope of our study and its implications. In this study, we were not able to estimate the potential savings of various other related cost outcomes due to lack of data, for example outcomes pertaining to presenteeism, short-term sickness benefit transfers or municipal care services. Also, our study design did not allow for estimating the costs of disability pensions since well-being does not involve a diagnosis of which disability pensions are based, but other studies have documented inverse associations between well-being and intentions to retire early [54] as well as risk for disability retirement [55]. Other studies have reported the benefits of well-being in terms of productivity [12, 13, 17] and retention [12, 13], which we were also not able to assess. Our estimated costs of sickness benefit transfers only include the costs of compensating individuals for long-term absence from work due to illness, but do not cover the actual value of lost productivity for these same individuals. In other words, the scope of covered costs included in our study is by no means exhaustive, and from a societal perspective, our final results would represent an underestimate in terms of reductions in costs associated with increases in mental well-being. Future research is needed to replicate the results of the study using 1) a larger sample with a higher response rate, 2) data with longer follow-up, and 3) the aforementioned outcomes that were not available to us. Also, further analyses that capture some of the broader financial benefits of promoting mental well-being to sectors outside the health care sector are warranted.

## Conclusion

The results of the present study support and expand prior findings in that population mental well-being predicts future expenditure (expressed in USD PPP) pertaining to healthcare costs and sickness benefit transfers. Higher mental well-being (on a continuous scale) in 2016 was associated with lower costs in 2017, i.e., each point increase in mental well-being was associated with lower healthcare costs ($− 42.5, 95% CI = $− 78.7, $− 6.3) and lower costs in terms of sickness benefit transfers ($− 23.1, 95% CI = $− 41.9, $− 4.3) per person in 2017. The relationship was linear, implying that moving from low to high mental well-being across the entire continuum is associated with considerable cost savings. The results were robust when considering differences in sociodemographics, psychiatric and somatic health status, as well as health behavior. Estimates cover the cost outcomes included in this study and pertain to the short-term reductions in costs associated with increases in mental well-being. Our estimates must therefore be considered conservative from a societal perspective. Our results suggest that investing in the promotion of population mental well-being, while being desirable in its own right, would have the additional benefit of curbing a wide range of costs. Universal mental health promotion initiatives that focus on moving all segments of the population towards higher levels of mental well-being could free up resources and reduce costs in the short term, potentially being cost neutral, as well as generate cost savings for society in the longer term.

## Figures and Tables

**Table 3:**
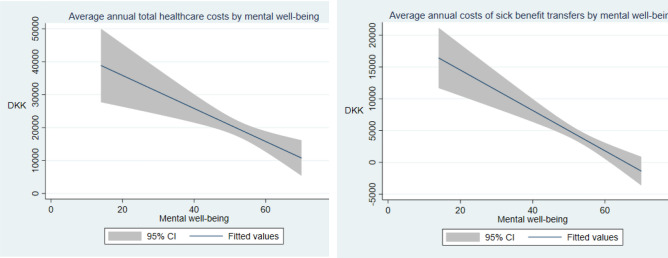
Unadjusted fitted lines for healthcare costs and sickness benefit transfers^a^ in 2017 (reported as DKK) by mental well-being^b^ (measured in 2016) among Danish adults aged 16 + years

All outcomes are 2017 costs only. DKK = Danish Krone (official currency of Denmark), 1DKK = USD$0.13 PPP

^a^The sample was restricted to individuals aged 16–64 years old (*N* = 1839)

^b^Warwick–Edinburgh Mental Well-being Scale (WEMWBS), range 14 (low)–70 (high)

## Appendix 1: information on healthcare and non-healthcare costs components

**Table:**

Register-based cost studies	The tracking of individual contacts with the healthcare system over time and across institutions is made feasible by an anonymized unique personal identification number (CPR number). A resident’s CPR number is granted at birth or upon immigration and is included in all national registers. Statistics Denmark encrypts the CPR number before releasing data for research [56, 57]
General Practitioners (GPs) and specialists	Unit costs for contacts with the primary care sector were estimated based on the fee paid by the government to the healthcare professionals (General Practitioners and specialists). The data on contacts with the primary care sector was stratified from the Danish National Health Service Register (NHSR)). The NHSR contains information on doctor and patient centered data Data on services for GPs and specialists comprises more than 200 individually priced services. The prices are agreed upon between the Organization of General Practitioners and the Danish regions. Data on contacts with patients and type of service delivered are reported to the NHSR as all GPs are linked to a uniform computer system. [56, 58]
Hospitalizations and outpatient services	The National Patient Register (Danish: Landspatientregisteret (LPR)) was used to obtain the costs for hospitalization and outpatient treatment [59] The LPR includes both administrative and clinical data. The administrative data is patient centered. As soon as a person has been examined or hospitalized, the hospital records a series of information about the patient: The person’s CPR number, background information on causes leading to hospital contact, etc. Clinical data relates to diagnosis and treatment procedures. Here, the LPR adapts the International Classification on Diseases, version (ICD-10). The National Patient Register includes all full-time admissions, emergency room contacts, and outpatient contacts for each CPR number respectively. Each treatment for a similar condition is linked to a rate that represents the average cost of a treatment course. We used these Diagnosis Related Groups charges (DRG) for admissions and Danish outpatient charges (DAGS) for outpatient contacts as unit cost estimates [60]. Admissions related to normal births, sterilization or healthy companions (the system registers when a patient needs a companion during appointments) were not included
Prescription medicines	The Danish National Prescription Registry (DNPR) was used to estimate the unit costs of prescription medicines. The DNPR holds information on prescription drugs acquired at Danish pharmacies. We applied the market price as a unit cost estimate [64]. Out-of-pocket payments were deducted from the total cost, thereby generating a cost outcome that pertains strictly to government healthcare expenditure
Sickness benefit transfers (long-term)	The LPR was used to extract relevant information on sickness benefit transfers. The Danish register includes information on sickness benefit transfers (long term) when individuals are absent from work due to sickness for at least 31 days, i.e., absence is not registered for periods less than 31 days (short term). Sickness benefit transfers (long term) are estimated based on the weekly number of hours absent from work and respective salary [61]–[63]. Analyses on sickness benefit transfers were only applied to the subgroup of the study sample who fall in the working-age population (range of 16–64 years)

## Appendix 2: results in DKK

See appendix Tables 3, 4.

**Table 4:** Marginal effects of a one-point change in mental well-being (measured in 2016, continuous scale^a^) on healthcare costs and sickness benefit transfers (2017 costs, reported as DKK) per person among Danish adults aged 16 + years

	Model 1		Model 2	
	Marginal effect	95% CI	Marginal effect	95% CI
	Healthcare costs			
Mental well-being	− 313.6	− 559.2, − 68.0	− 317.8	− 588.6, − 47.0
	Sickness benefit transfers^b^			
Mental well-being	− 215.2	− 360.6, − 69.9	− 172.6	− 313.1, − 32.1

DKK = Danish Krone (official currency of Denmark), 1DKK = USD$0.13 PPP

Model 1 adjusted for age, sex, migration background, education, marital status, employment status, income, and 2016 costs. Model 2 adjusted for all the aforementioned covariates as well as chronic conditions, number of psychiatric conditions, and physical activity

^a^Based on the 14-item Warwick–Edinburgh Mental Well-being Scale (WEMWBS), range 14–70

^b^The sample was restricted to individuals aged 16–64 years old (*N* = 1839)
